# Solid-state Lidar with wide steering angle using counter-propagating beams

**DOI:** 10.1038/s41598-023-34199-4

**Published:** 2023-09-24

**Authors:** Yuxuan He, Qiang Wang, Xu Han, Zhonghan Wang, Wenpu Geng, Yuxi Fang, Zhongqi Pan, Yang Yue

**Affiliations:** 1 Institute of Modern Optics, Nankai University, Tianjin, 300350 China; 2 Angle AI (Tianjin) Technology Co. LTD, Tianjin, 300350 China; 3 Department of Electrical and Computer Engineering, University of Louisiana at Lafayette, Lafayette, LA 70504 USA; 4 School of Information and Communications Engineering, Xi’an Jiaotong University, Xi’an, 710049 China

**Keywords:** Integrated optics, Optoelectronic devices and components

## Abstract

In a solid-state photonics-based Lidar, all essential components can be integrated into a silicon chip. It is simple and effective to use a tunable laser source to implement Lidar’s beam steering. However, how to effectively increase the steering angle in a small wavelength tuning range is usually a key challenge due to the limited material and waveguide dispersion. In Silicon-on-insulator waveguide, we design a novel solid-state Lidar using two trans-electrical (TE) polarized beams counter-propagating towards each other. Two corresponding output beams from just a single grating coupler (GC) can be seamlessly combined to double the beam steering angle. Furthermore, a low-priced solid-state Lidar is designed for TE polarized beams counter-propagating towards each other by using wavelength division multiplexed laser array.

## Introduction

For robotics, self-driving vehicles, weather research, drones and 3D sensing system, Lidar is a critical component^1^. Using photons as the sensing medium, Lidar is able to capture information with a wide range of degrees of freedom, including wavelength, space, amplitude, time, phase, and polarization. Thus, Lidar is an ideal tool for sensing over a wide range^2–4^. The traditional Lidar consists of disconnected components, which have high cost, big footprint, complex design, and intrinsic instability. All components of the solid-state photonics-based Lidar are integrated into a single-crystal silicon substrate, resulting in a small size, high reliability, modest cost, and low power consumption^5–9^. Scanning the space is a crucial task for Lidar, which requires an efficient beam steering mechanism. Among a wide variety of beam steering mechanisms, wavelength tuning of the source is always one of the simplest and most effective methods to achieve beam steering^10–12^. A grating coupler (GC) can be etched from the Silicon-on-insulator (SOI) waveguide. The angle of beams diffracted from the GC are adjusted by altering the wavelength of the light source^13, 14^. As SOI materials have limited dispersion, wavelength tuning methods face the challenge of effectively increasing the steering angle within a narrow wavelength adjusting range.

Two beams, one in the trans-magnetic (TM) polarization and the other in the trans-electrical (TE) polarization, are seamlessly combined to double the beam steering angle^15^. Nevertheless, this requires additional polarization manipulation, and two different GC designs for the TM and TE polarizations. In this work, a new structure is proposed and designed to double the beam tuning angle by using counter-propagating beams with the same polarization. This design can significantly simplify the whole on-chip Lidar architecture^16–19^.

This paper is structured as follows: we present in “Lidar with tunable laser” an integrated Lidar structure that is capable of doubling the beam steering angle when only TE-polarized light is used. Two counter-propagating TE signals generate two diffracted beams from a single GC^20^. In order to seamlessly merge two beams, GC parameters need to be selected properly. As the result, beam steering angle is thus doubled. This idea is first demonstrated by using a tunable laser source (TLS) with the wavelength range from 1.5 to 1.6 μm. In “Discussion”, we replace the TLS with a four-channel wavelength division multiplexing (WDM) laser array operating in the C-band. The wavelengths are 1.55 μm, 1.57 μm, 1.59 μm, and 1.61 μm, respectively. The simulation results show that our design can indeed work with the WDM laser array. Using two counter-propagating inputs, eight output beams are formed, and the beam-steering angle is thus doubled. In “Conclusion”, the GC design is further optimized so that the Lidar can work near 1310 nm. The light source is a four-channel WDM laser array working in the O-band, and the wavelengths are 1.271 μm, 1.291 μm, 1.311 μm, and 1.331 μm, respectively. This O-band WDM laser array has been suitable for the short-reach optical communication modules, leading to a significant cost advantage. Similarly, its beam-steering angle is doubled by investing two counter-propagating inputs.

## Lidar with tunable laser

**Figure 1:**
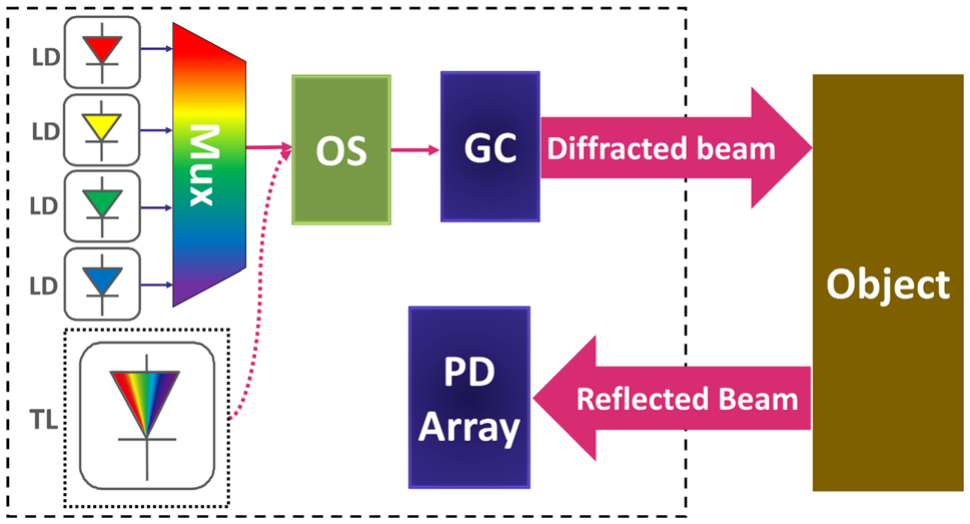
Block diagram of our proposed solid-state Lidar system. *LD* laser diode, *Mux* multiplexer, *TL* tunable laser, *OS* optical switch, *GC* grating coupler, *PD* photodiode.

Figure 1 illustrates the configuration of our proposed solid-state Lidar system. The wavelength of the light source can be either continuously tunable or discrete. TE polarization is selected for the input beams, and the output beams are diffracted into the surrounding environment. For continuously tunable light source, a double steering angle can be achieved when two diffracted beams are combined. For the input light source with *N* discrete wavelengths, 2*N diffracted beams are generated. The diffracted beam will be reflected by the objects in the environment, and detected by a photodiode array^21–23^. An integrated system can be developed by integrating all the components within the dashed box for saving cost and simplifying the device structure.

### Principle and methods

**Figure 2:**
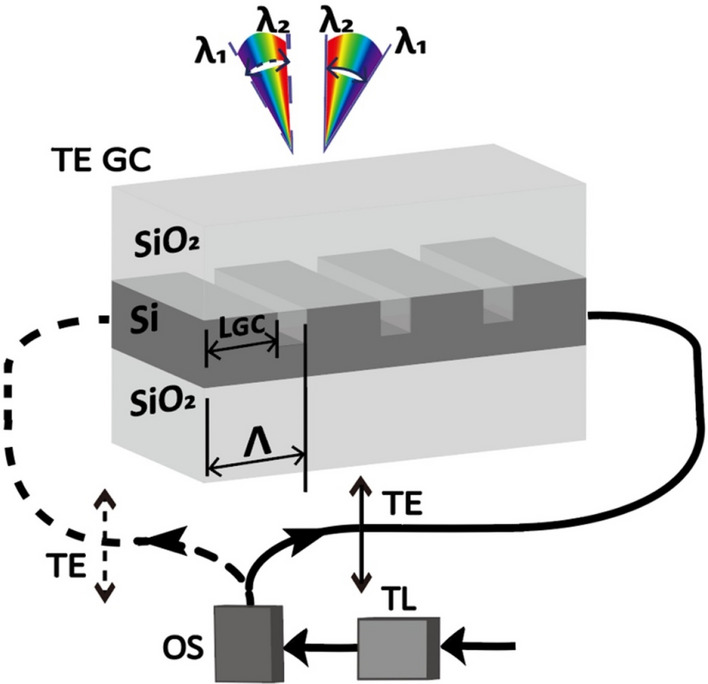
Configuration of a GC based on counter-propagating TE polarized beams to double the beam steering angle.

The configuration of the on-chip Lidar system is shown in Fig. 2. A GC and an optical switch are integrated on a single substrate. It is common for light sources to produce linearly polarized signals, which are coupled into the SOI waveguide with the elementary TE mode. The TE mode is chosen, as it has a larger effective refractive index and a smaller bending loss. In the description below, a TLS is chosen as the light source. The same operating principle holds for the case of using a WDM laser array as the light source.

According to the need, the optical switch can fully manipulate the beam and steer it into one of the two counter-propagating directions^24^. The beam is directed towards the left path during the first period. For the light propagating from the left to the right in the GC, the wavelength is swept from *λ*_*1*_ to *λ*_*2*_ and the first beam is diffracted from the GC. The light is directed to the right path by the optical switch during the second period. For the light propagating from the right to the left in the GC. In reverse, the wavelength is swept from *λ*_*2*_ to *λ*_*1*_, and the second beam is diffracted from the GC. There is an obvious mirror symmetry between the first beam group and the second beam group. If the diffraction angle of *λ*_*2*_ is close to the vertical line, a seamless combination of two beams can be attained, and this results in a doubled beam-steering angle. For a continuous operation, the procedure above is repeated.

The designed GC parameters are optimized for our proposed Lidar. The light is diffracted out of the SOI waveguide by shallowly etching the GC. The following principle is implemented to form an unseamed beam with doubled steering angle: at one end of the tuning range (*λ*_*1*_), the angle between the diffraction angle of the TE mode and the vertical line is the largest; as the wavelength is tuned from this end to the other end of the wavelength sweeping range (*λ*_*2*_), the diffraction angle gradually gets close to the vertical line and eventually goes through the vertical line; at the longer wavelength end (*λ*_*2*_), the TE mode diffracts on the contrary side of the vertical line with a narrow diffraction angle. To meet these requirements, the following GC parameters are chosen: the width of the SOI waveguide is 670 nm and the height is 295 nm. A proportion of the SOI (length *L*_*GC*_) remains intact at the grating period of 610 nm, while the other is etched by 135 nm to realize the optical GC. The filling factor *f* is defined as the ratio between the *L*_*GC*_ and the grating period *Λ*. *f* is also a crucial parameter and it is set to 0.52. Utilizing two counter-propagating TE beams with individual GC and selecting these parameters can double the beam-steering angle.

The proposed structure can perform one-dimensional beam steering. By duplicating the proposed configurations of the GC and integrating multiple GCs in TE mode on the same substrate, one can achieve two-dimensional beam-steering. In this way, wavelength tuning can be used to guide the beam in one direction, while optical phase array (OPA) can be used to steer the beam in the other dimension. With this design, the beam steering range will be greatly enhanced when wavelength tuning is employed.

Only a single GC is shown in Fig. 2 for its simplicity. As part of the actual implementation, multiple GCs can be further integrated into an OPA. By using a switch to direct the light, one can create two counter-propagating beams with TE polarization. The input signal can be split into multiple GCs using a binary tree structure, which is composed of numerous cascading broadband Y-splitters^25^. The beams can be steered in the direction perpendicular to the GC by regulating the phase between adjacent GCs. Beam steering in two dimensions can be achieved with this method.

### Calculations in theory

The purpose of this section is to determine the considerable parameters of the TE GC using theoretical analysis when a TLS is used. The TLS operates within the wavelength range between 1.5 and 1.6 μm. This wavelength band is typically used for long-haul optical communication. The TLS in this wavelength range is mature and cost-effective. The GC parameters are adjusted to make sure that a seamless combination of two diffracted beams' output angles could be achieved.

Considering a shallow-etched GC with an underlying structure whose physical size is a lot smaller than the optical wavelength, Eq. (1) reveals the effective index of this shallow-etched GC as a result of effective mode theory (EMT)^26, 27^:

1 $${n}_{eff}=f{n}_{H}+(1-f){n}_{L}$$

where *n*_*H*_ refers to the effective index of the SOI's intact portion (295 nm), *n*_*L*_ represents the effective index of the SOI's etched portion (160 nm).

**Figure 3:**
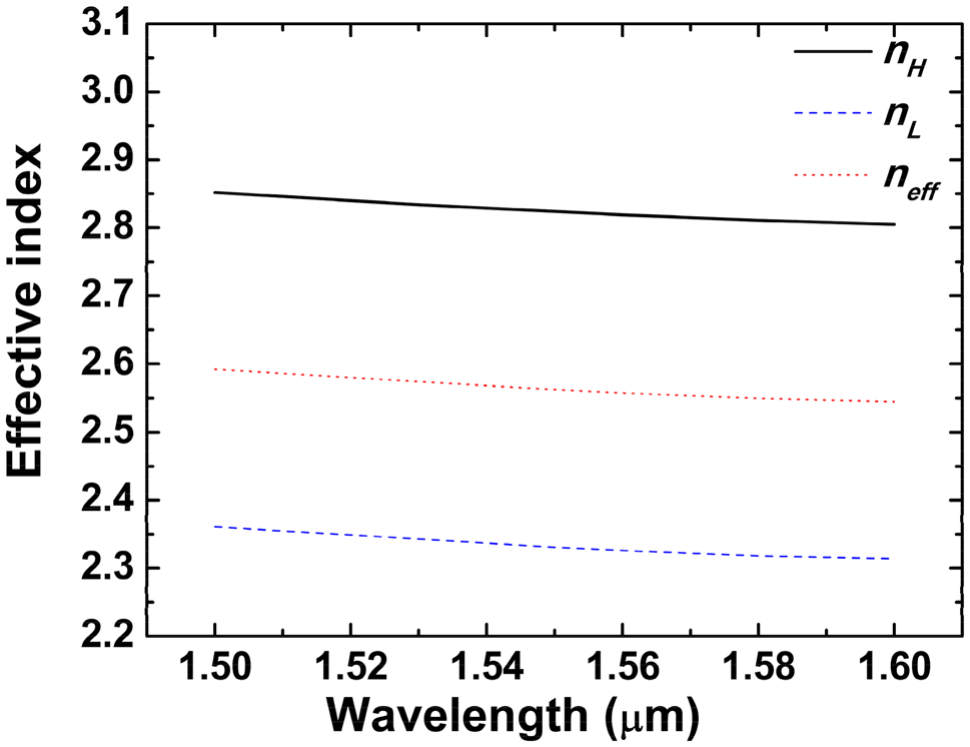
Numerical results of *n*_*eff*_, *n*_*H*_, and *n*_*L*_ for TE mode.

Utilizing an eigenmode solver for finite difference, the *n*_*H*_ and *n*_*L*_ values for TE modes are computed within the 1.5–1.6 μm wavelength range, as illustrated in Fig. 3. In order to calculate *n*_*H*_, the size of buried oxide (BOX) SOI waveguide, which is intact, is 670 nm × 295 nm. The BOX SOI waveguide has a dimension of 670 nm × 160 nm during *n*_*L*_ calculation.

Here is the equation showing how the diffraction angle is related to the effective index *n*_*eff*_ based on Eq. (2):

2 $$k{n}_{{e}_{ff}}=k{n}_{c}\mathit{sin}\varphi +2\pi q/\Lambda$$

where *n*_*c*_ refers to the refractive index of the SiO_2_ cladding, *k* = *2π / λ*, and *q* represents the order of those diffraction beams in our design, which is set to 1. The output angle *φ* is stipulated as the angle between the normal of SOI surface and the diffracted beams in the far field. Note that light propagates along a left-to-right direction in Eq. (2). When the light is propagating towards the left, the sign of the output angle is reversed. Based on this consideration, *φ* will be positive when the TE-polarized beam is propagating from left to right and negative when the TE-polarized beam is propagating in the opposite direction.

To satisfy our design requirement, a diffraction beam should exit along a vertical line and its diffraction angle is zero:

3 $$\mathrm{sin}\varphi =0, \varphi =0$$

As follows, Eq. (2) is rewritten for the TE polarized mode:

4 $$2\pi {n}_{{e}_{ff}} / \lambda =2\pi q/\Lambda$$

**Figure 4:**
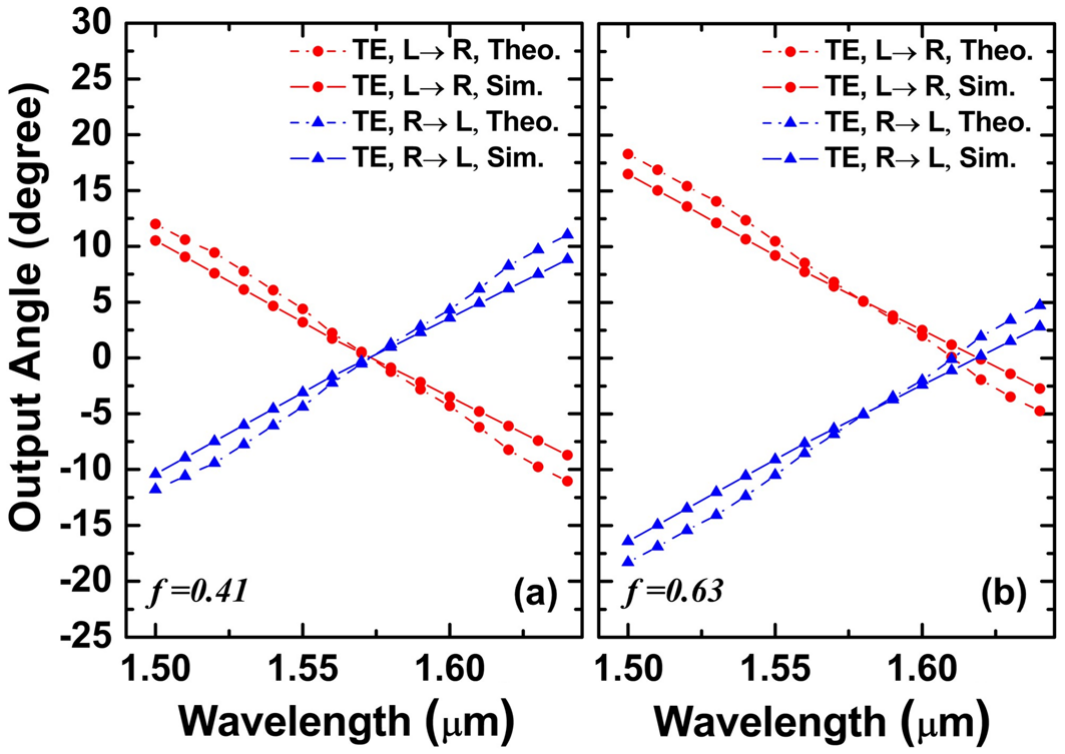
Theoretical and numerical 3D results of two counter-propagating TE beams’ output angles for (**a**) *f* = 0.41 and (**b**) *f* = 0.63.

As a result of the above considerations, the following parameters are selected: for the TE GC, the filling factor *f* is 0.52 and the period is *Λ*_*TE*_ = 610 nm. The numerical results for the TE mode are shown in Fig. 3. Figure 4 depicts two representative results for *f* = 0.41 and *f* = 0.63, respectively. Based on the *f*-scan results, it is set to 0.52 to satisfy the condition that the output angle is right along the 0° vertical line with a 1.6-μm incident light source. In the next section, a detailed theoretical explanation will be given of the diffraction angles generated by two output beams.

The frequency domain power transmission of the GC can be calculated with the following formula:

5 $$T(f)=\frac{\frac{1}{2}{\int }_{\text{monitor }} \mathrm{Re}({\varvec{P}}(f))\cdot d\mathbf{S}}{\text{ source power }(f)}$$

where *T*(*f*) is the normalized transmission as a function of frequency, ***P***(*f*) is the Poynting vector, and *dS* is the surface norm. The normalization of the continuous wave does not affect the result of the normalized transmission *T*(*f*).

To save the cost, one can adopt an approach to share one TLS among a number of integrated Lidars. Through the use of a multimode interference splitter (MMI), TLS can split its light output into several light paths. In this way, each GC with counter-propagating beam can be supported by a discrete-wavelength light source. This approach can significantly reduce the cost compared with using TLS for a Lidar system.

### 2D simulation results

A numerical simulation of the model is conducted using the finite difference time domain (FDTD) method. Using this way, Maxwell's equation can be solved in the time domain to model the nanophotonic devices. By using the FDTD simulation, the beam’s propagation angle *φ* for different wavelengths can be calculated when the device under consideration is injected with a wideband light source. The simulation is carried out on a laptop with Intel Core i5-8250U CPU, which is running at 1.60 GHz clock. By using a workstation with more advanced CPUs, a significant reduction in running time can be achieved. The running time is also dependent on the type of FDTD simulation. For 2D simulation, its running time is under one minute. But for 3D simulation, its running time can be up to 2.5 h. To reduce the simulation time as much as possible, we first perform 2D FDTD in order to establish a rough estimate of the critical GC parameters. Our next step is to carry out a 3D simulation to acquire an accurate estimate.

One needs a boundary condition to solve the Maxwell equation. As the back reflection at the edge of the simulation area will affect the numerical results, a perfect matching layer (PML) is chosen as the periphery in order to diminish the back reflection. A structure similar to Fig. 2 is used as the simulation model, and TE-polarized light source is chosen. The silicon GC resides between silica materials on the top and the bottom, and a broadband laser source is initially launched into the SOI waveguide without etching. For the wavelength range under consideration, both the waveguide dispersion and the material dispersion are included in the simulation. The near-field diffracted electromagnetic field is collected by placing a monitor on top of the silica layer.

**Figure 5:**
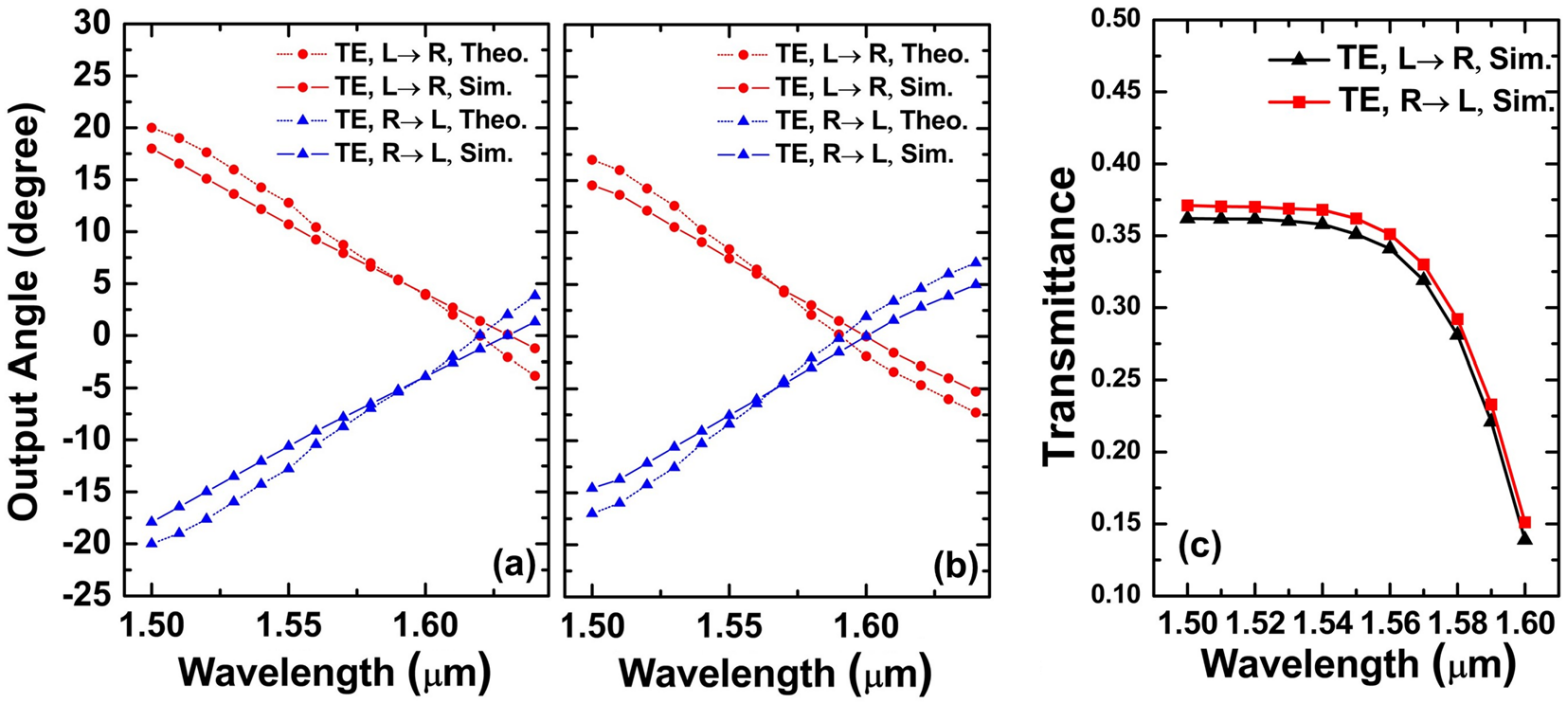
Theoretical and numerical results of two counter-propagating TE beams’ output angles in (**a**) 2D simulation and (**b**) 3D simulation; (**c**) transmittance for both TE beams under the 3D simulation.

A theoretical analysis and numerical simulation utilizing 2D-FDTD are conducted to obtain the corresponding two sets of output angle value, as shown in Fig. 5a. In the theoretical analysis, a slab waveguide is assumed as in the 2D-FDTD simulation, in which the width dimension of the waveguide is infinite. *n*_*H*_ and *n*_*L*_ are then calculated. According to the simulation result, two TE beams have same diffraction angle and propagation direction at *λ*_*2*_ = 1.63 μm. If the incident light has a wavelength of 1.63 μm, the output angle is right on the vertical line. As the wavelength of the light source changes from *λ*_*1*_ = 1.53 μm to *λ*_*2*_ = 1.63 μm, the diffraction angle of the TE beam propagating to the left decreases from 13.8° to 0°, and gradually approaches the vertical line. The diffraction angle of the beam propagating from the right to the left also changes from −13.8° to 0° accordingly. These two beams are jo ined at a vertical line, resulting in a 27.6° beam steering angle over the 100-nm wavelength tuning range. Although there is a small deviation between the theoretical and simulation results, they are basically consistent, which shows that the design principle proposed is valid.

When performing 2D-FDTD simulations, we assume the waveguide to be a slab rather than a stripe. It is not considered that the refractive index varies along the width of the waveguide. The result of this simplification is that theoretical analysis and numerical results deviate from each other. The approximate range of the parameters is determined through 2D-FDTD simulations due to its extremely short running time.

### 3D simulation results

We analyze through the theoretical computation and the numerical simulation using 3D FDTD, which is very complicated and runs over long time. It is time consuming to optimize a design over a wide range of parameters. Therefore, 3D FDTD is used to fine-tune the parameters of the GC after the 2D simulation.

As can be seen in Fig. 5b, the output angle obtained through theoretical computation and numerical simulation using 3D FDTD is presented. The starting plane here is based on the 3D FDTD simulation. A monitor is placed right outside the top Silica layer to collect the near-field diffracted electromagnetic field. Then, the far field of the diffracted beam can be calculated using Fresnel–Kirchhoff diffraction formula. The wavelength range of the incident light is from 1.5 to 1.6 μm. The *φ* value indicated the maximum spot of the electrical field emitted from the GC in the far field. As seen, the simulation results are in good agreement with the theoretical analysis. This demonstrates that the theory can predict the output angle at different wavelengths with high accuracy. Between *λ*_*1*_ = 1.5 μm and *λ*_*2*_ = 1.6 μm, the right-propagating TE beam can be controlled between 14.53° and − 0.06°, and the diffracted angle for left-propagating TE beam is between − 14.55° and 0.03°. As the incident light wavelength is 1.6 μm, its output angle just passes through the vertical position. The beam steering range can be increased to 29° with a tuning range of approximately 100 nm by propagating TE beams from opposite directions through the waveguide.

**Figure 6:**
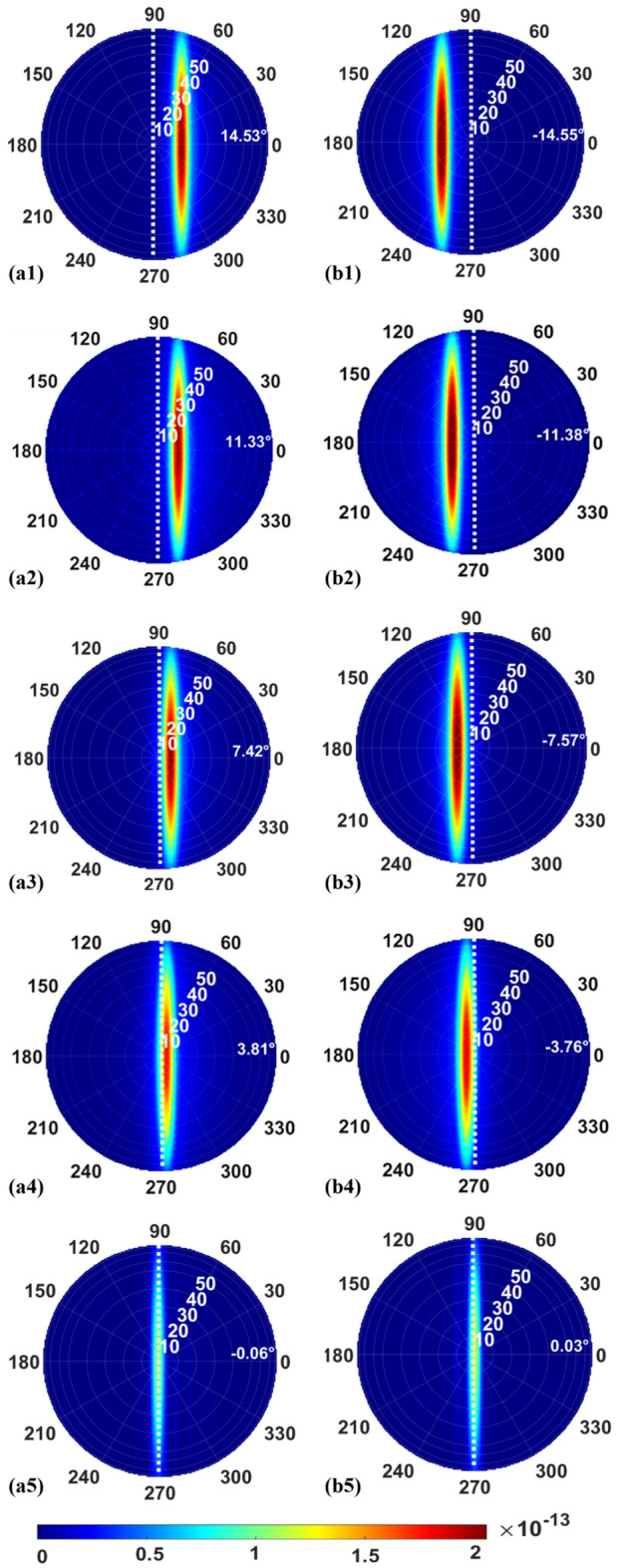
Beam profiles in the far-field for the TE beams propagating leftward at (**a1**) *λ*_*1*_ = 1.5 μm, (**a2**) *λ*_*2*_ = 1.525 μm, (**a3**) *λ*_*3*_ = 1.55 μm, (**a4**) *λ*_*4*_ = 1.575 μm, (**a5**) *λ*_*5*_ = 1.6 μm, and rightward at (**b1**) *λ*_*1*_ = 1.5 μm, (**b2**) *λ*_*2*_ = 1.525 μm, (**b3**) *λ*_*3*_ = 1.55 μm, (**b4**) *λ*_*4*_ = 1.575 μm, (**b5**) *λ*_*5*_ = 1.6 μm, respectively.

As shown in Fig. 6, the Fresnel–Kirchhoff diffraction formula is used to calculate the far-field beam profiles for five wavelengths of 1.5 μm, 1.525 μm, 1.55 μm, 1.575 μm and 1.6 μm. In terms of describing the beam emanating from a point source in three-dimensions, the spherical coordinate system is ideal. When the wavelength increases, the output angle for the TE mode decreases and is closer to 0°. Lastly, the output angle crosses the vertical line. There is an approximate 14.5° difference for the input wavelengths 1.5 μm and 1.6 μm. By seamlessly combining two output beams formed by a couple of counter-propagating inputs diffracted from a single GC, the total beam steering angle is doubled. For the GC, adding apodization of the grating period in the direction of signal propagation will further optimize its spot size^28, 29^. Adding top cladding or bottom mirror can enhance the upward emission efficiency, which is one of the most significant factors^30^.

Figure 5c presents the transmittance of the TE beam. It is determined as the ratio between the energy of the up-propagating beam and the energy injected into the GC^30–32^. When the wavelength is 1.5 μm, the transmittance of the TE mode is the largest, which is about 37%. Over the wavelength range of 1.5–1.55 μm, its transmittance is almost constant. Within the wavelength range of 1.55–1.6 μm, the transmittance decreases with a steep gradient. When the wavelength is 1.6 μm, the transmittance of the TE mode is the smallest, which is about 14%. The transmittance decrease can be clearly observed from the far-field intensity profile in Fig. 6. As there is an obvious wavelength dependence, the large transmittance difference will cause uneven energy emission and reduce the overall device performance. One solution is to adjust the output power of the TLS to make up for the reduction of transmittance in the long wavelength regime. Another solution is to substitute a WDM laser array for the TLS which can alleviate this problem. More details will be provided in the next section.

## Discussion

### Lidar with WDM laser array in C band

Solid-state Lidar systems can also be equipped with a WDM laser array. The Lidar using WDM as the light source has the characteristics of fast measurement speed, less data processing and low cost. Like the existing multi-line Lidar, it can be applied to security protection, terrain mapping, intelligent driving assistance and other fields. The outputs from multiple lasers are combined through a WDM multiplexer. Several beams are diffracted through a GC whose output angles are influenced by their wavelengths. There are a few advantages for replacing TLS with WDM laser arrays. Firstly, WDM laser arrays are widely used in the optical communication, leading to a significant cost advantage. Secondly, simultaneous operation of multiple lasers leads to a much higher detection rate. For a Lidar using TLS, only one data point can be collected at a specific time. For a Lidar using WDM laser arrays, all lasers can send the light sources simultaneously. A WDM demultiplexer can separate the returning signal based on different wavelengths, and a PD array can detect those signals simultaneously. Thirdly, one can select the wavelengths so that the diffracted beam does not propagate along the vertical direction to the GC. This will reduce the variation of transmittance as discussed in “Lidar with tunable laser”. Fourthly, the output power of the individual laser within the laser array can be individually and precisely controlled to compensate any wavelength dependence of the transmittance.

**Figure 7:**
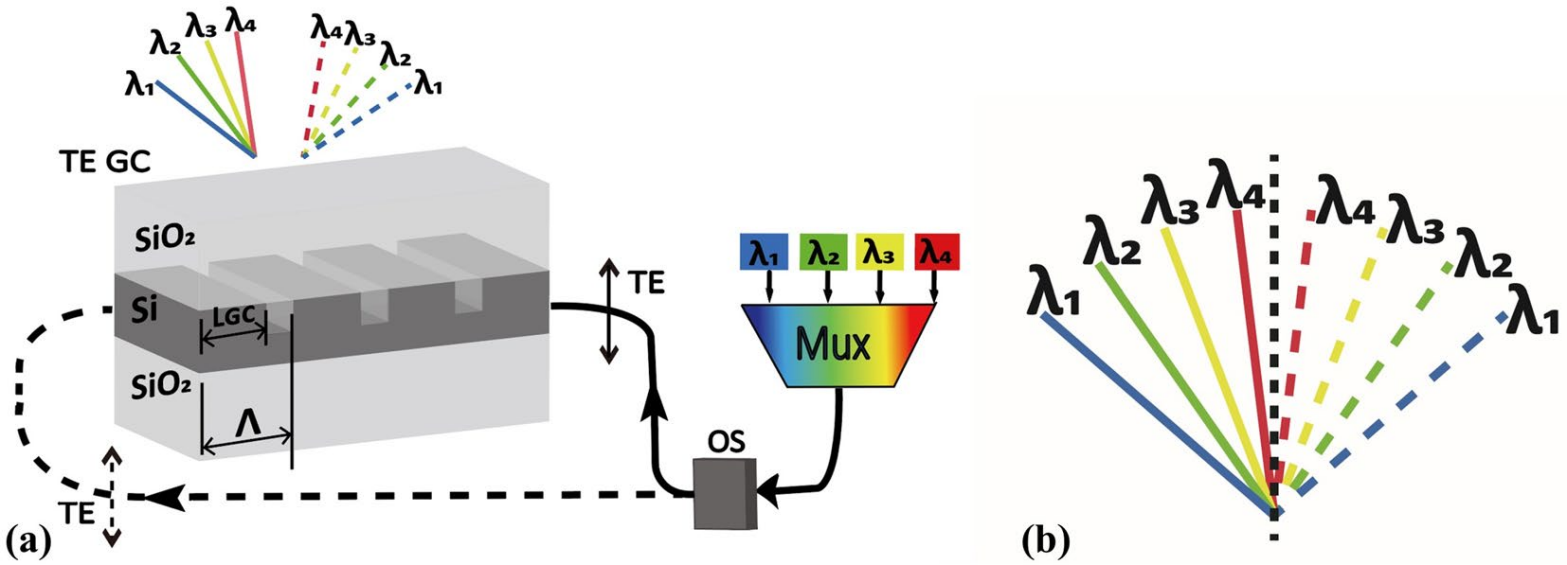
(**a**) Grating coupler (GC) and system configuration utilizing counter-propagating TE polarized beams; (**b**) diagram of the diffracted output angles for the TE polarized WDM beams produced by the single GC.

The designed GC structure is depicted in Fig. 7a. In this design, four wavelengths (*λ*_*1*_ = 1.55 μm, *λ*_*2*_ = 1.57 μm,* λ*_*3*_ = 1.59 μm, *λ*_*4*_ = 1.61 μm) are chosen in the C band, which align with the coarse WDM wavelength grid. The optical switch changes the propagating direction of the combined beams through the single GC. As a result, a total of eight beams are diffracted out by a GC of counter-propagating inputs in TE polarization^32–37^. The diagram of the diffracted output angles for the TE polarized WDM beams is shown in Fig. 7b. Unlike the structure proposed in the previous section, this new structure allows for a 7/3 times increasement of the viewing angle by utilizing the TE polarized lights.

A shallow-etched GC with optimized parameters is chosen for the TE mode, which is utilized to diffract the beam away from the SOI waveguide. The critical GC parameters for using discrete lasers, like etched depth and grating period, are different from using a continuously tuneable laser. The SOI waveguide is designed with a width of 600 nm. A portion of the Si layer (length *L*_*GC*_) is unetched with a height of 220 nm in a grating period, and the remaining part is etched by 100 nm. The filling factor *f* is another critical factor, which can be calculated as the ratio between *L*_*GC*_ and *Λ*. *Λ* and *f* are optimized to achieve our design target as discussed above.

The GC can diffract all the light beams of four wavelengths. There are four diffracted beams per direction of propagation, each with a different output angle determined by its wavelength. When the beam is guided into the GC from a reverse direction, the direction of the diffracted beam is reversed. The angle between two neighbouring output beams (*φ*_*adj*_) is around 3.1°. There is the largest angle far from the 0° vertical line at 1550 nm (*λ*_*1*_). When the wavelength is 1610 nm (*λ*_*4*_), the smallest diffraction angle (*φ*_*min*_) is 1.55°, which is nearest to the vertical line. To ensure that eight beams can produce uniformly distributed angles, *φ*_*min*_ should be approximately one half of *φ*_*adj*_ (*φ*_*min*_ ≈ 0.5*φ*_*adj*_). The GC is designed accordingly to satisfy this requirement.

Figure 7a illustrates the layout of the solid-state Lidar transmitter system. The TE mode is selected for the outputs of the WDM laser array and a WDM multiplexer is used to combine the outputs. The output signal is then routed either in the right or left direction by an optical switch. It is divided into two stages of operation. During the first phase, the light is pointed to the left direction and produces four beams diffracted by the GC. During the second procedure, the optical switch routes the signal to the right pathway and then produces four beams generated in the reversed direction. Four diffracted TE polarized beams and one GC, combined with two stages, can more than double the viewing angle. It is worth noting that our design is not just for four channels. By using a similar design approach, we can scale the number of channels beyond four.

**Figure 8:**
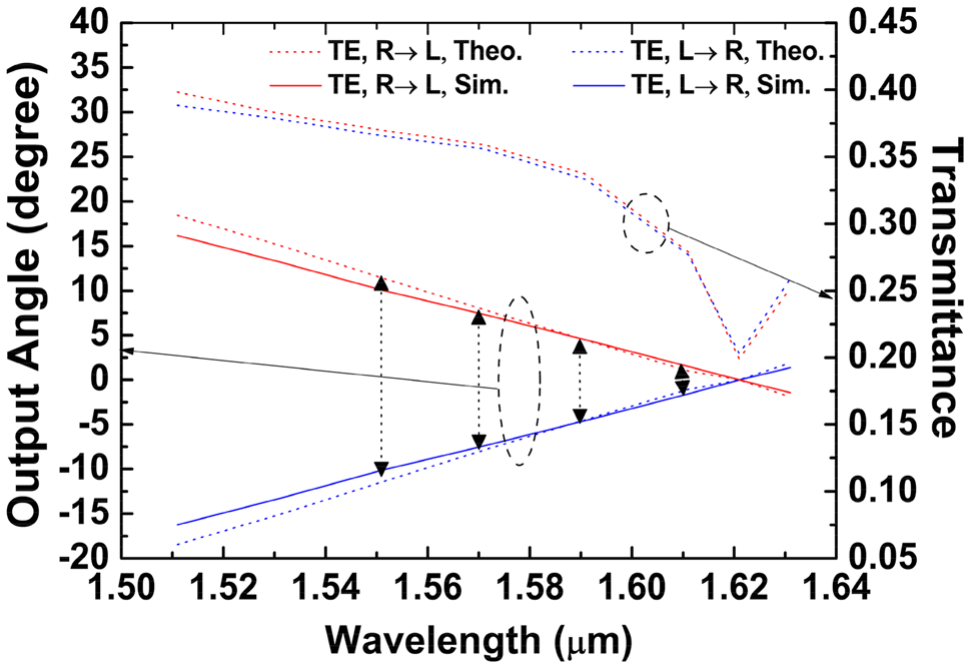
Transmittance and output angles of four beams. Arrows mark the locations of four input wavelengths.

As shown in Fig. 8, the transmittance drops significantly when *φ* is close to zero. When the input light is generated using a TLS, the diffraction angle *φ* should be close to zero as much as possible. Otherwise, two beams generated from two counter-propagating beams cannot be seamlessly combined. When a WDM laser array with multiple discrete wavelengths is used, one only needs to satisfy the condition of *φ*_*min*_ ≈ 0.5*φ*_*adj*_ to guarantee the diffracted beams are uniformly distributed. Thus, we can avoid the undesired situation where the diffracted beam propagates in the direction vertical to the GC. As discussed earlier, it is much easier to control the power of the individual laser to compensate the variation in transmittance.

The determination of key GC parameters for the TE mode is based on theoretical analysis. We modify those parameters to make sure *φ*_*mi*n_ ≈ 0.5*φ*_*adj*_, and the angles of the eight output beams can be evenly distributed. The effective refractive index of the GC is calculated using Eq. (1), and the output angle is derived using Eq. (2).

Next, the design is verified through numerical simulation utilizing 3D-FDTD. The filling factor and the period of the TE GC are *f* = 0.35 and *Λ* = 450 nm. The theoretical analysis as well as the numerical simulation result based on 3D-FDTD are presented in Fig. 8. The simulation is in good agreement with the theory for the TE mode, and this shows our design method is still feasible when using discrete lasers. When the wavelength is *λ*_*1*_ (1550 nm), the largest output angle is approximately 10.9°, and there is around 3.1° separation between two adjacent beams. While the wavelength is 1.610 μm, the output angle is the smallest, which is approximately 1.55°. Asymmetric distribution of the output angles along the vertical line occurs when the light comes into the GC from different directions. Figure 8 also displays the transmittance of these beams. As seen, the transmittance curve is relatively smooth and changes slowly. For the TE mode, the maximum transmittance is around 38% and the minimum transmittance is about 28%.

**Figure 9:**
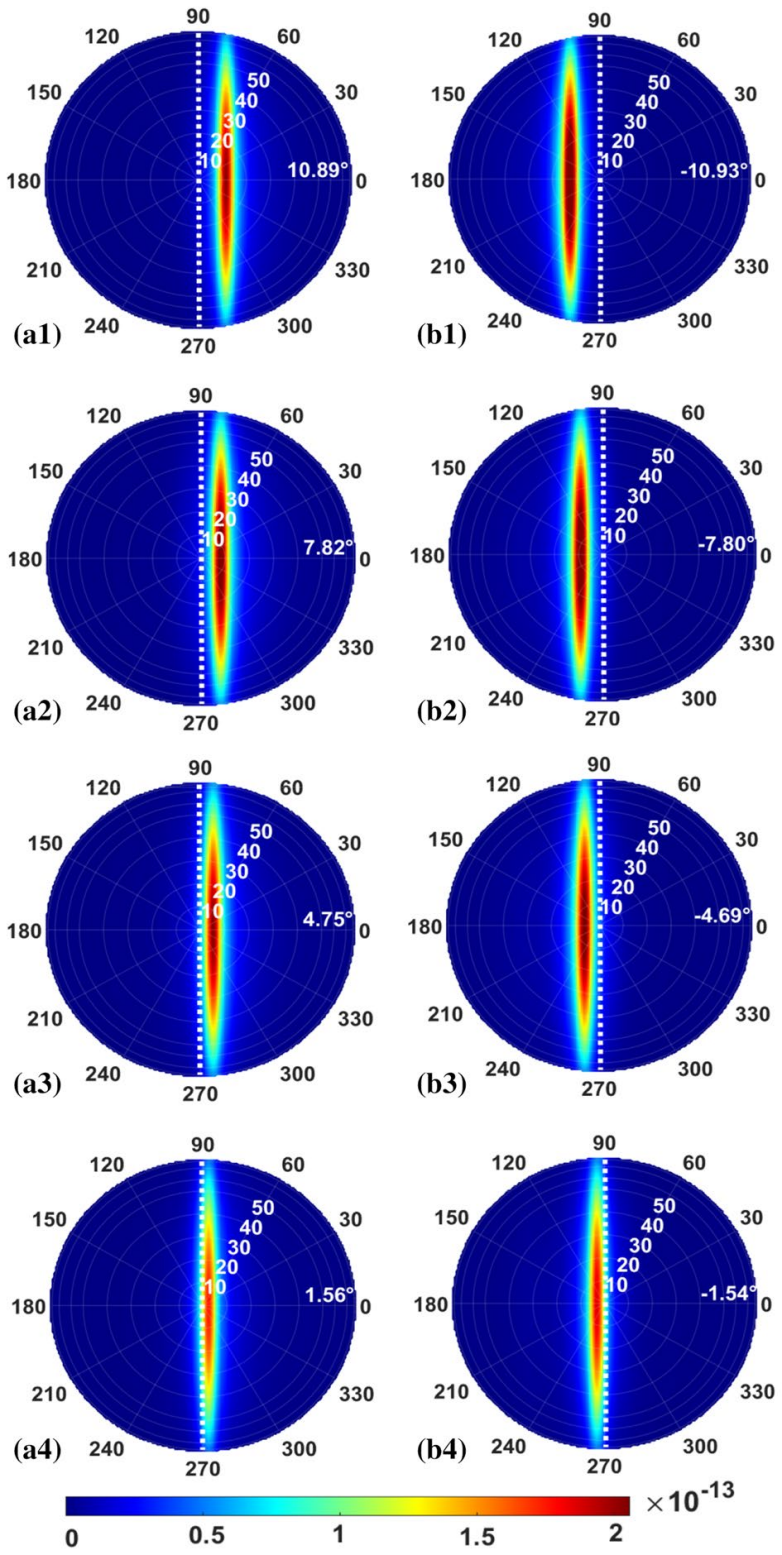
Beam profiles in the far-field for the TE beams propagating leftward at (**a1**) *λ*_*1*_ = 1.551 μm, (**a2**) *λ*_*2*_ = 1.571 μm, (**a3**) *λ*_*3*_ = 1.591 μm, (**a4**) *λ*_*4*_ = 1.611 μm, and rightward at (**b1**) *λ*_*1*_ = 1.551 μm, (**b2**) *λ*_*2*_ = 1.571 μm, (**b3**) *λ*_*3*_ = 1.591 μm, (**b4**) *λ*_*4*_ = 1.611 μm, respectively.

Figure 9 shows the far-field intensity distribution of the input TE-polarized WDM source. The Fresnel–Kirchhoff diffraction formula is also used to calculate the distribution of the far-field beam. With an increase in wavelength, the output angle of the TE mode decreases. The largest output angle of the TE mode is approximately 10.9°. By using the design with counter-propagating beams, a 22° angle of view can be achieved. Also, one can notice that the far-field intensity profile does not change significantly among different wavelengths. This demonstrates one advantage of using the WDM laser array as the laser source.

### Lidar with WDM laser array in O band

In the previous section, we demonstrate that one can replace the TLS with the WDM laser array in the same wavelength range. The advantages of using WDM laser array as the light source is also discussed. In this section, we extend this design to the WDM laser array near 1310 nm (O band). For the short-reach optical transceiver widely applied in the web-scale data center, the direct-detection scheme is the preferred solution due to its reduced energy consumption and lower cost. The chromatic dispersion is a detrimental impairment to the direction-detection scheme. Since the zero-dispersion wavelength in a single mode fiber is around 1.31 μm, all short-reach optical transceivers work in the O band. The CWDM4 transceiver has the largest volume among different optical transceivers. Four wavelengths are *λ*_*1*_ = 1.271 μm, *λ*_*2*_ = 1.291 μm, *λ*_*3*_ = 1.311 μm, *λ*_*4*_ = 1.331 μm. The solid-state Lidar can also be equipped with the WDM laser array and WDM photodiode array in the O band. This will lead to a significant cost advantage by leveraging the large volume of CWDM4 transceiver.

**Figure 10:**
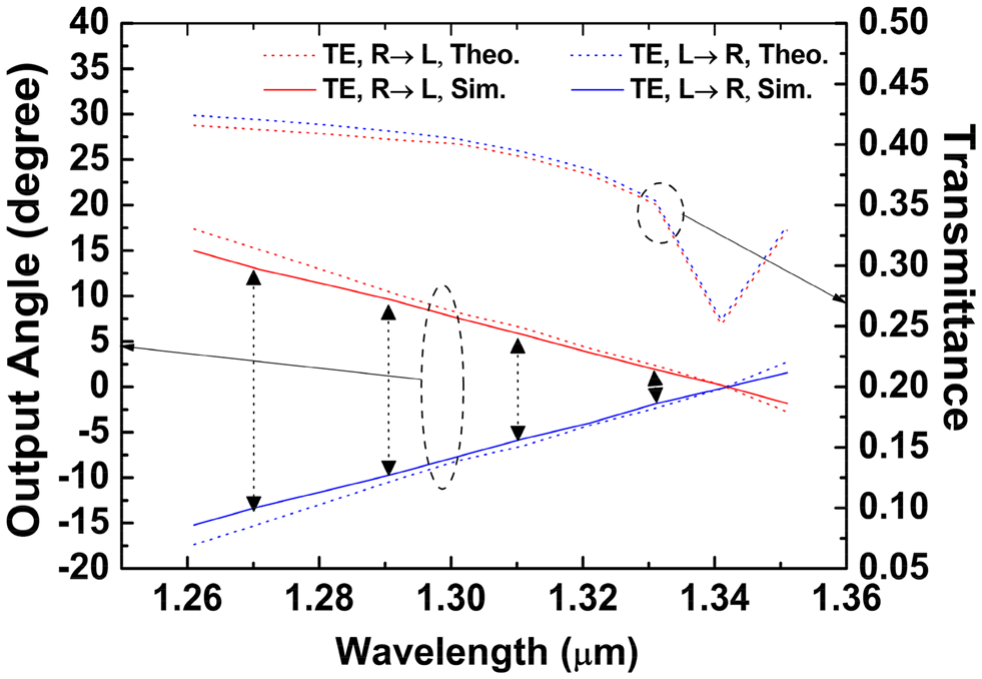
Transmittance and output angles of four beams. Arrows mark the locations of four wavelengths.

In this design of GC for O-band wavelength, the period *Λ* of 520 nm corresponds to the filling factor *f* of 0.53. We use Eqs. (1) and (2) to acquire the theoretical results. 3D FDTD method is used to acquire the simulation outcomes. As shown in Fig. 10, there is good agreement between the simulation results and the theoretical results. The output angles satisfy the requirement of (*φ*_*min*_ ≈ 0.5*φ*_*adj*_), and a uniform distribution of eight beams is achieved. Both values are set at 224 nm. The transmittance curve is also displayed in Fig. 10 for eight TE output beams. As seen, the maximal transmittance is ~ 0.42 and the minimal transmittance is ~ 0.35. The usage of WDM laser array in the O band can reduce the transmittance variation as well. The weaker grating strength can be achieved by optimizing the thickness of the SiO_2_ upper cladding layer and the SiO_2_ lower cladding layer.

**Figure 11:**
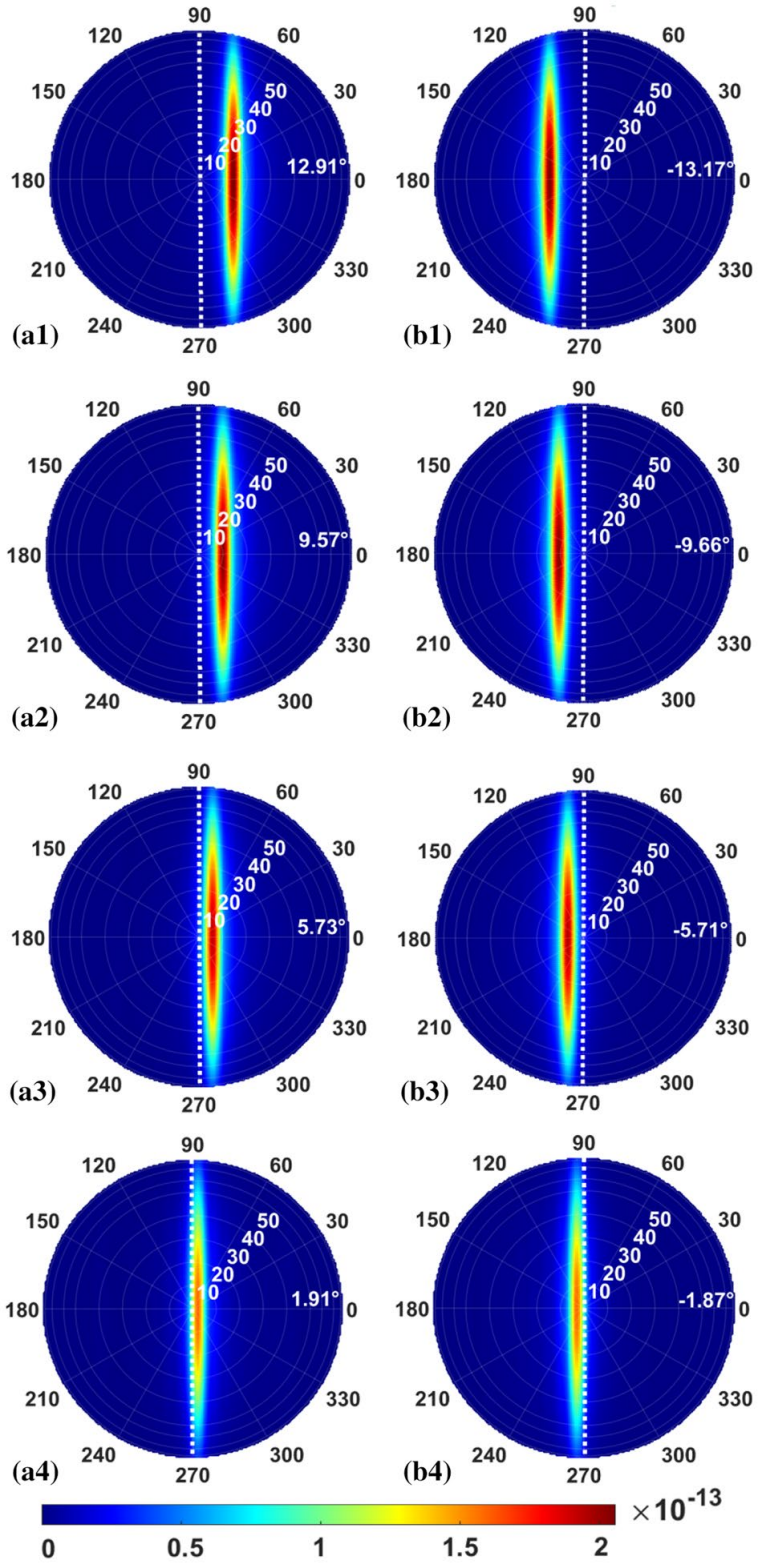
Beam profiles in the far field for the TE beams propagating leftward at (**a1**) *λ*_*1*_ = 1.271 μm, (**a2**) *λ*_*2*_ = 1.291 μm, (**a3**) *λ*_*3*_ = 1.311 μm, (**a4**) *λ*_*4*_ = 1.331 μm, and rightward at (**b1**) *λ*_*1*_ = 1.271 μm, (**b2**) *λ*_*2*_ = 1.291 μm, (**b3**) *λ*_*3*_ = 1.311 μm, (**b4**) *λ*_*4*_ = 1.331 μm, respectively.

As shown in Fig. 11, the beam profiles in the far field have been determined using the Fresnel–Kirchhoff formula. As seen, the first group of four beams is generated when the light is propagating in the GC from the left to the right. By reversing the propagation direction in the GC, the second group of four beams is generated. These two beam groups are mirror symmetric to each other along the vertical axis. The combined output from the WDM laser array propagating through the GC in reverse directions can provide a total of 26° view angle.

## Conclusions

To realize the solid-state integrated Lidar using photonic technology, wavelength tuning can be implemented as an efficient and convenient beam steering method. The TLS wavelength range, however, severely limits the beam steering angle. In this paper, a new design is presented to achieve double steering angle. Two output beams can be generated from a single GC by using counter-propagating TE polarized light. When the appropriate GC parameters are selected, the output beams of the GC will propagate in the reverse direction. The two beams can be seamlessly merged to increase the beam steering angle by twofold. Based on theoretical analysis and simulations, the feasibility of our design has been demonstrated.

On this basis, a CWDM laser array with four discrete wavelengths can achieve larger scale and lower cost. We further optimized the CWDM wavelength to achieve a larger viewing angle and higher efficiency. This novel concept is numerically verified for the CWDM laser array working in C band or in the O band. The transmittance variation can be reduced by using CWDM laser array as the light source.

## Data Availability

The datasets used and/or analysed during the current study available from the corresponding author on reasonable request.
